# Exercise-Conditioned Endothelial Progenitor Cell-Exosomes Preserve Cerebral Blood Flow and Alleviate Acute Ischemic Brain Injury in Hypertensive Mice

**DOI:** 10.3390/life16040623

**Published:** 2026-04-08

**Authors:** Shuzhen Chen, Smara Sigdel, Gideon Udoh, Brandon Xiang Yu, Jinju Wang

**Affiliations:** Department of Biomedical Sciences, Joan C. Edwards School of Medicine, Marshall University, Huntington, WV 25755, USA

**Keywords:** exercise, EPC-EXs, acute ischemic stroke, neuroinflammation, p38

## Abstract

Exosomes (EXs) mediate intercellular communication in the tissue microenvironment. We previously demonstrated that endothelial progenitor cell-derived exosomes (EPC-EXs) from exercised mice protect neurons and cerebral endothelial cells from hypoxia- and hypertension- induced injury ex vivo, suggesting their therapeutic potential in hypertensive ischemic injury. Here, we investigated whether exercise-conditioned EPC-EXs (ET-EPC-EXs) confer protection against acute ischemic injury. Hypertensive transgenic mice were divided into donor and recipient groups. Donor mice underwent treadmill exercise to generate ET-EPC-EXs. Recipient mice was subjected to middle cerebral artery occlusion and received ET-EPC-EXs via tail vein injection (2 × 10^8^/100 μL saline) two hours after stroke onset. Cerebral blood flow (CBF) was assessed, and brains were collected on day two for histological and molecular analyses. Our data showed that ET-EPC-EXs were robustly taken up by cerebral cells, predominantly in the penumbra in the ipsilateral hemisphere. ET-EPC-EXs reduced cell death and microglia activation and restored tight-junction proteins. Moreover, ET-EPC-EX treatment preserved CBF and improved sensorimotor function on day two post-stroke. Mechanistically, ET-EPC-EXs suppressed p38 activation, accompanied by reduced matrix metalloproteinase-3 and cytochrome c levels in the ipsilateral brain. Collectively, these findings demonstrate that EPC-EXs from exercise mice improve sensorimotor functions and confer protection in hypertensive ischemic brain injury, likely through attenuation of neuroinflammation and preservation of vascular integrity via modulation of the p38 signaling.

## 1. Introduction

Stroke remains one of the leading causes of death and long-term disability in the United States. The burden of ischemic stroke is disproportionately elevated in individuals with hypertension. Compared with normotensive patients, hypertensive individuals are more likely to develop larger infarcts [1], more severe cerebral edema [2], accelerated blood–brain barrier (BBB) breakdown [3], and poorer neurological recovery [3]. These hypertension-driven vulnerabilities underscore the need for therapeutic strategies that directly target the cerebrovascular system during the acute phase of ischemic injury.

Exercise is well established as an effective nonpharmacological approach for improving vascular health and reducing stroke risk. Prior studies demonstrate that exercise promotes angiogenesis in ischemic brain regions by upregulating endothelial nitric oxide synthase and increasing the production of endothelial progenitor cells (EPCs) in mice and Sprague-Dawley rats [4,5]. Exercise also confers neuroprotection in ischemic stroke by enhancing neuronal plasticity and upregulating neurotrophic growth factor expression [6,7,8]. Increasing evidence suggests that the benefits of exercise may be mediated by exercise-induced changes in the secretome of circulating reparative cells, including bone marrow-derived EPC extracellular vesicles (EVs) [9,10].

Exosomes (EXs), a major class of small EVs, have attracted increasing interest as cell-free therapeutics due to their ability to transfer bioactive proteins, lipids, and nucleic acids that modulate signaling in recipient cells. EPC-derived exosomes (EPC-EXs) are of particular interest because they directly participate in vascular repair processes. EPC-EXs have been shown to promote angiogenesis [11], support vascular repair [12], and accelerate recovery from hindlimb ischemia [13]. Interestingly, exercise has recently been revealed to improve cardiac fibrosis through stimulating cardiac EPC-EX secretion [14]. Our recent work demonstrated that exercise increases the reparative capacity of bone marrow-derived EPC-EXs [9]. Specifically, exercise-preconditioned bone marrow-derived EPC-EXs (ET-EPC-EXs) can improve endothelial cell survival and attenuate neuronal oxidative damage in vitro [9,15]. However, whether ET-EPC-EXs can mitigate acute ischemic injury in the hypertensive brain has not been systemically investigated.

Mitogen-activated protein kinases (MAPKs) are key regulators that translate extracellular stress signals into intracellular responses governing survival, inflammation, and metabolism [16]. Among MAPKs, p38 plays central roles in ischemic injury and hypertension-related cerebrovascular dysfunction [16,17]. Inhibition of p38 MAPK has been shown to reduce angiotensin II-induced vascular injury [18] and ameliorate aortic stiffening [19]. Activation of the p38 signaling pathway is reported to negatively regulate BBB integrity through modulating the expressions of tight junction proteins such as zonula occludens-1 (ZO-1) and increasing metalloproteinase activity [20]. Inhibition of the p38 pathway protects against ischemic brain injury in rodent animals [21]. Together these findings suggest that the p38 pathway is implicated in early BBB breakdown and neurovascular inflammation in stroke.

In the present study, we used a hypertensive transgenic mouse model subjected to middle cerebral artery occlusion (MCAO) to evaluate the neurovascular protective effects of ET-EPC-EXs administered during the acute post-stroke period. We assessed cerebral blood flow recovery, sensorimotor function, neuroinflammation, and tight-junction protein preservation. We also examined whether ET-EPC-EXs modulate the p38 signaling pathways during the early ischemic phase in hypertension. We focused on the acute phase of ischemic stroke, as effective neurovascular protection during this stage is critical for improving survival and provides a necessary foundation for subsequent long-term functional and cognitive assessments.

## 2. Materials and Methods

### 2.1. Animals and the Treadmill Exercise Protocol

Human renin hypertensive transgenic mice (129S/SvEv-Tg; Alb1-Ren; 2Unc/CofJ) from the Jackson Laboratory were used in this study. All experimental mice were inbred and housed under 12 h light/dark cycles at the Marshall University Animal Facility. All mice were given complete free access to normal chow and water. Mice of both sexes in approximately equal numbers were studied. All protocols were approved by the Marshall University Laboratory Animal Care and Use Committee, and all experiments were conducted by the Guide for the Care and Use of Laboratory Animals (published by the National Research Council) and reported in compliance with ARRIVE guidelines.

The Alb1 mice (male and female; 7–8 weeks old) were subjected to an eight-week moderate treadmill exercise [15]. In brief, all exercise mice were adapted to the treadmill (Columbus Instruments, Columbus, OH, USA), running for five days before the beginning of treatment. During day 1 of training, mice ran at 5 m/min for 30 min, and each following day of training, the speed was increased by 1 m/min and the time by 10 min. Following training, the exercise mice group underwent 60 min of running at 10 m/min, 5 days/week for eight weeks.

### 2.2. Preparation and Characterization of EPC-EXs from Exercised Mice (ET-EPC-EXs)

After exercise, all mice were sacrificed, and their bone marrow was collected. EPCs were isolated and cultured from bone marrow [22]. In brief, the bone marrow was flushed out of the femurs and tibias using a 1 mL syringe with 1 mL PBS. Then the bone marrow suspension was gently layered over 2 mL of gradient medium (Hisopaqu-1083, Cat #10831; Sigma-Aldrich, Inc. St. Louise, MO, USA) for centrifugation at 800× *g* for 30 min at 4 °C. The mononuclear cells in the interface layer were collected in a new 15 mL tube and washed with 1 mL PBS by centrifugation at 400× *g* for 5 min at 4 °C. Cell pellets were resuspended with EC basal medium-2 (EBM-2) supplemented with EPC growth factors (Lonza, Morristown, NJ, USA) and seeded into a tissue culture T25 flask. After 2 days in culture, non-adherent cells were removed by washing with PBS. After that, the culture medium was changed every 2 days. After 5 days of culture, EPC medium was changed to serum-free EBM-2 medium supplemented with growth factors (Lonza, Morristown, NJ, USA) for 2 days to stimulate EX release.

EXs were collected from the conditional medium using the ultracentrifuge method [23]. In brief, the culture medium was collected and centrifuged at 300× *g* for 15 min, and the supernatant was centrifuged at 2000× *g* for 20 min to remove cell debris. The resulting supernatant samples were centrifuged at 20,000× *g* for 70 min to remove microvesicles, followed by ultracentrifugation at 170,000× *g* for 90 min to pellet EXs. All collected EXs were characterized by nanoparticle tracking analysis (NTA) and Western blot with exosome-specific markers, Tsg101 and CD81, as reported [24]. For in vivo studies, the collected EXs were resuspended in filtered saline.

### 2.3. Middle Cerebral Artery Occlusion (MCAO) Surgery and ET-EPC-EX Infusion

The hypertensive Alb1 mice were subjected to MCAO surgery to induce an ischemic stroke model. Briefly, mice were anesthetized in a chamber with 2.5% isoflurane. Surgery was performed under 1–2% continuous isoflurane, and the animal’s body temperature was maintained using a heating pad. Adequate sedation was confirmed by tail pinch for all surgery procedures. The common carotid artery was exposed and ligated distal to the bifurcation, then the external carotid artery was ligated and cut for exposure of the internal carotid artery. A suture was placed under the carotid internal artery and lightly lifted to prevent blood backflow from the head. Then, a small incision was made on the common carotid artery between the ligation and the carotid bifurcation. A 7-0 silicone-coated filament suture with a rounded head was inserted through the small incision and advanced into the internal carotid artery until resistance was detected (about 10 mm distal to the bifurcation). The suture was left in place with a ligation for permanent MCAO. Pain and discomfort were minimized by using buprenorphine (3.25 mg/kg, s.c). Two hours after MCAO onset, mice (*n* = 9/group) were randomly assigned to receive 100 uL ET-EPC-EXs (2 × 10^8^ EXs in 100 μL saline) or vehicle (saline) treatment by intravenous tail vein injection. The EXs collected from the bone marrow EPCs of one donor mouse were injected into one recipient mouse. To track the injected EXs, a red fluorescence dye, PKH 26 (2 μM, Sigma-Aldrich), was used to label the ET-EPC-EXs according to the manufacturer’s instructions and our previous report [9]. In brief, EX pellets were resuspended with 2 μM PKH26 dye in 1 mL filtered PBS for 5 min at room temperature, then 1 mL FBS was added to stop staining, followed by ultracentrifugation. The labeled EXs were resuspended in saline for tail vein injection into the hypertensive mice subjected to MCAO surgery. Two days after EX infusion, mice were euthanized with an i.p. injection of pentobarbital (150 mg/kg), and brain samples were collected.

### 2.4. Sensorimotor Function Evaluation

The sensorimotor deficits were assessed by the adhesive removal test as previously reported with slight modifications [25]. Mice were tested before (day −1) and after stroke (day 0), and 2 days after MCAO surgery. All mice were subjected to a 3-day training to ensure they learned to tear the tape off before being assigned to experiments. On the test day, all mice were acclimated to the test room for 30 min before testing. In brief, a small piece of adhesive tape (0.3 cm × 0.4 cm) was placed on the mouse’s paw. The time taken to remove the tape was recorded. The cutoff time was 180 s. Three trials were conducted to get an average value for each animal. The interval time was 5 min.

### 2.5. Cerebral Blood Flow Measurement

A laser speckle imaging system (RFLSI III, RWD Life Science, Sugar Land, TX, USA) was used for monitoring cerebral blood flow at different time points: before MCAO surgery (day −1), after MCAO surgery (day 0), and 2 days after the EX-treatment (day 2). CBF in the ischemic region decreased to less than 30% of the baseline and was considered a success. In brief, after anesthesia, the mouse was placed in a prone position on a thermostatic mouse plate. The skin and mucous membrane of the head were separated to keep the skull fully exposed. Saline was instilled on the skull to maintain a wet condition. The laser speckle imaging system was focused on the mouse skull to obtain a clear color map. The cortical blood flow of both cerebral hemispheres was recorded. The region of interest was set. The value of the region of interest represents the blood flow in the selected region. CBF data at different time points was expressed as a percentage of the baseline.

### 2.6. Cresyl Violet (CV) Staining

Forty-eight hours after EX treatment, all mice were sacrificed with euthanasia solution. Brains were transcardially perfused with ice-cold PBS and 4% paraformaldehyde (PFA), followed by 4% PFA overnight and in 4% PFA plus 30% sucrose for 3 days. Then, coronal sections (20 μm) were obtained using a cryostat. For infarct volume quantification, five sections spanning the rostral-caudal extent of the lesion were selected for CV staining as previously described [26]. The lesion area was quantified using the NIH Image J system (Version: 1.54r). Infarct volume was calculated by dividing the lesioned area of the ipsilateral hemisphere by twice the total area of the contralateral hemisphere.

### 2.7. TUNEL Assay of Cell Apoptosis Analysis in the Brain Tissue

In situ apoptosis of brain tissues on day 2 post-MCAO-surgery was measured by terminal deoxynucleotidyl transferase (TdT) dUTP Nick-End Labeling (TUNEL) assay kit (Roche, Basel, Switzerland) according to the manufacturer’s instructions. In brief, brain slices (20 μm) were mounted on gelatin-coated slides and permeabilized with 0.1% TritonX-100/0.1% sodium citrate for 2 min. The samples were then washed and incubated with a freshly prepared TUNEL reaction mixture in an incubator for 60 min at 37 °C in the dark. Cell nuclei were stained with 4′, 6-diamidino-2-phenylindole (DAPI, 1 μg/mL; Wako Pure Chemical Industries Ltd., Richmond, VA, USA). Tissue samples were examined under a fluorescence microscope (Nikon, Eclipse E600, Shinagawa, Japan). TUNEL+ cells were counted at three microscopic fields and averaged. An average of four sections from rostral to caudal represented the data for one mouse.

### 2.8. Immunofluorescence Analysis in the Brain Tissue

To determine whether ET-EPC-EXs affect tight junction protein expression in the brain, immunofluorescence was used. We analyzed the expressions of tight junction proteins occludin and ZO-1 in the brain 2 days after ET-EPC-EX treatment. Brain coronal sections were incubated with CD31 (1:50; Novus Biologics, Centennial, CO, USA; NB600-1475), NeuN (1:100, EMD Millipore, St. Louis, MO, USA, MAB377), GFAP (1:200; Thermo Fisher, Waltham, MA, USA; PA1-10019), IBA-1 (1:500, Fujifilm, Santa Ana, CA, USA; 019-19741), ZO-1 (1:100, Abcam, Cambridge, MA, USA; zb221547), occludin (1:100, Abcam; ab216327) antibody overnight at 4 °C. Next, brain sections were reacted with Cy3 (red) or FITC (green) conjugated secondary antibodies (1:250; Invitrogen, Carlsbad, CA, USA) for 2 h at room temperature in the dark. The positive cells in the peri-infarct area of each section were imaged using a fluorescence microscope (Olympus XP70; Boston Industries, Inc., Walpole, MA, USA) equipped with an ACCU-SCOPE Excelis MPX-20RC cooled color microscopy camera (20 Megapixels; New York Microscope Company, Hicksville, NY, USA). To analyze co-localization, uptake of ET-EPC-EXs was categorized by markers such as CD31^+^PKH26^+^ for microvessels, NeuN^+^PKH26^+^ for neurons, GFAP^+^PKH26^+^ for astrocytes, and IBA-1^+^PKH26^+^ for microglia. Tight junction protein expression in microvessels was identified by CD31^+^occludin^+^ or CD31^+^ZO-1^+^. GFAP-labeled cells were counted from randomly selected fields and IBA-1 fluorescence intensity per field was analyzed in the peri-infarct area of the ipsilateral hemisphere in four mice from each group. For each mouse, four sections were evaluated and three random microscopic fields per section were sampled. The data were analyzed using Image J software (NIH, Bethesda, MD, USA, Version: 1.54 r) and Prism Graphpad 10 (San Diego, CA, USA).

### 2.9. Western Blot Analysis

The proteins of the brain tissue were extracted using lysis buffer supplemented with a complete mini protease inhibitor tablet. A bicinchoninic acid (BCA) assay was performed to quantify protein concentrations. Protein lysates were electrophoresed through 6% or 10% SDS-PAGE gel or 4–12% Bis-Tris gel (Invitrogen) before being transferred onto polyvinylidene fluoride (PVDF) membranes, which were blocked with BSA buffer. Primary antibodies CD81 (1:200; Abcam; ab216130), Tsg 101 (2 ug/mL; Abcam; ab30871), p38 (1:1000; Cell signaling, Danvers, MA, USA; 9212), p-p38 (1:1000; Cell signaling; 4511), MMP3 (1:500, Abcam; ab52915), cytochrome c (Cyto-C), 0.25 ug/mL, Abcam; ab110325), and β-actin (1:4000; Sigma-Aldrich; A5441) were incubated at 4 °C overnight. The next day, membranes were washed and incubated with horseradish peroxidase-conjugated anti-rabbit or anti-mouse IgG (1:40,000; Jackson Immuno Research Lab, West Grove, PA, USA) for two hours at room temperature. Blots were imaged using chemiluminescent solutions in a ChemiDoc imager (Bio-Rad, Hercules, CA, USA) and analyzed using ImageJ 1.54f.

### 2.10. Statistical Analysis

Data are expressed as the mean ± SD. Comparisons between two groups were performed using an unpaired Student’s *t*-test. For multiple group comparisons, one-way ANOVA or two-way ANOVA followed by Tukey’s post hoc test was used. GraphPad Prism 10.5.0 was implemented for data analysis. For all measurements, *p* < 0.05 was considered statistically significant.

## 3. Results

### 3.1. ET-EPC-EXs Were Predominantly Incorporated in Brain Cells in Penumbra in the Ipsilateral Brain

**Figure 1:**
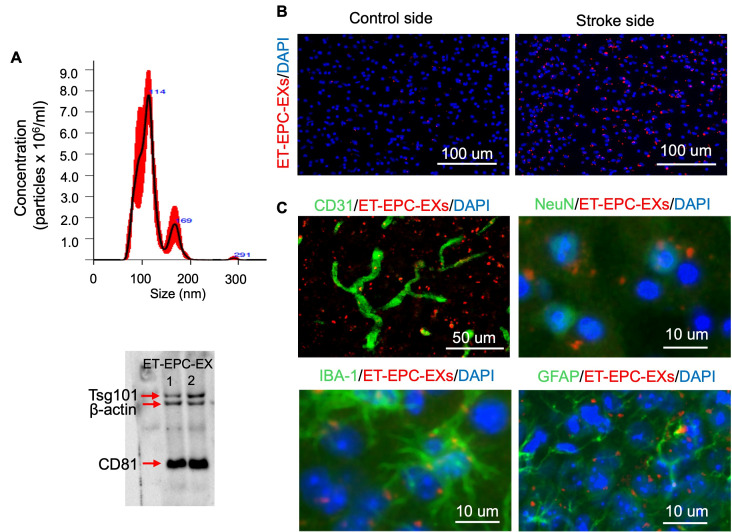
ET-EPC-EX incorporated with brain cells in hypertensive mice subjected to MCAO-induced ischemic stroke. (**A**) Representative plot showing size distribution and concentration of ET-EPC-EXs; *n* = 5/group. Western analysis of EX markers (CD81, Tsg101) in ET-EPC-EXs; *n* = 2/group. (**B**) EX distribution in the ipsilateral and contralateral hemispheres. Scale bar: 100 μm. (**C**) Representative images show co-localization with brain cells, CD31 for microvessels, NeuN for neurons, IBA-1 for microglia, and GFAP for astrocytes. EXs were labeled with red fluorophore PKH26. Scale bar: 10 μm or 50 μm; *n* = 3/group.

EXs isolated from the culture medium of bone marrow EPCs from exercised hypertensive Alb1 mice were characterized as reported in our previous study [27]. ET-EPC-EXs were in a cup shape and expressed the parent cell EPC-specific marker. They also expressed EX-specific markers such as CD81 and Tsg101 and had an average diameter less than 200 nm, as shown in Figure 1A. To determine whether ET-EPC-EXs can reach the brain, we labeled EXs with lipophilic fluorescence dye PKH 26 to track biodistribution in vivo. The labeled EXs were intravenously administered to the animals two hours after MCAO. Fluorescently labeled EXs were observed in the brain throughout both the ipsilateral and contralateral hemispheres. Of note, the fluorescence signal intensity was much stronger in the penumbra area in the ipsilateral hemisphere, compared to the contralateral hemisphere (Figure 1B), suggesting that ET-EPC-EXs were preferably home to the peri-infarct area to protect the brain against acute ischemic injury. Moreover, to determine the phenotypes of the labeled EX-positive cells, immunofluorescence staining was performed on the brain sections using antibodies against GFAP (for astrocytes), NeuN (for neurons), Iba1 (for microglia) and CD31 (for endothelial cells). As shown in Figure 1C, the labeled EX-positive cells (PKH26^+^) co-localized with NeuN, GFAP, Iba1 and CD31. These findings suggest that the intravenously injected ET-EPC-EXs could cross the BBB and be internalized by brain cells.

### 3.2. ET-EPC-EX Infusion Preserved CBF in Hypertensive Alb1 Mice After MCAO

**Figure 2:**
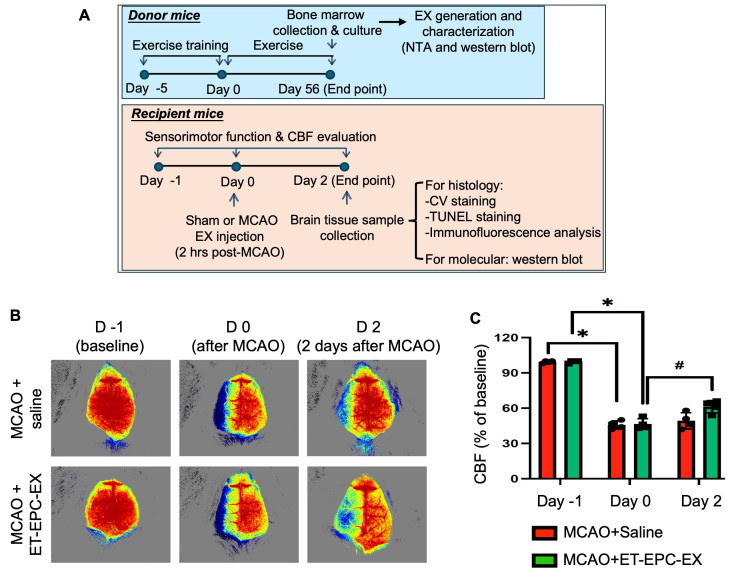
ET-EPC-EX preserved CBF in hypertensive mice subjected to MCAO-induced ischemic stroke. (**A**) Experimental timeline for the entire experiment procedures. Donor mice underwent exercise intervention for 8 weeks to generate exercise-conditioned EPC-EXs. Recipient mice underwent MCAO on day 0 and received ET-EPC-EXs or saline 2 h post-ischemia. CBF was assessed by laser speckle imaging system at baseline, post-occlusion, and day 2, followed by tissue collection for histological and molecular analysis. (**B**) Representative plots showing CBF in the mouse brain at different time points. Red/yellow indicates higher perfusion; blue indicates reduced flow. (**C**) The summarized data of CBF of the three groups at different time points. Quantification of CBF recovery expressed as percentage of baseline. * *p* < 0.0001; vs. saline at day −1; # *p* = 0.01; vs. saline at day 0; Data was analyzed using two-way ANOVA followed by Tukey test. Data was expressed as mean ± SD; *n* = 4 mice/group.

To determine whether ET-EPC-EX treatment affects collateral circulation establishment following ischemic stroke, we evaluated cortical perfusion using a laser speckle imaging system at baseline, after MCAO, and 48 h after EX treatment (Figure 2A). As shown in Figure 2B,C, all animals exhibited CBF reductions after MCAO, reflecting a successful occlusion. According to the day 2 data, mice that received ET-EPC-EXs displayed markedly restored perfusion compared with saline-treated mice, as indicated by increased red-yellow signal intensity on perfusion maps. In contrast, saline-treated mice showed persistent low blood flow perfusion, as reflected by extensive blue regions corresponding to sustained microvascular compromise. This finding suggests that ET-EPC-EXs act during the acute post-stroke window to support collateral circulation establishment and microvascular reperfusion.

### 3.3. ET-EPC-EXs Improved the Sensorimotor Function and Reduced Lesion Volumes of Hypertensive Alb1 Mice Subjected to MCAO

**Figure 3:**
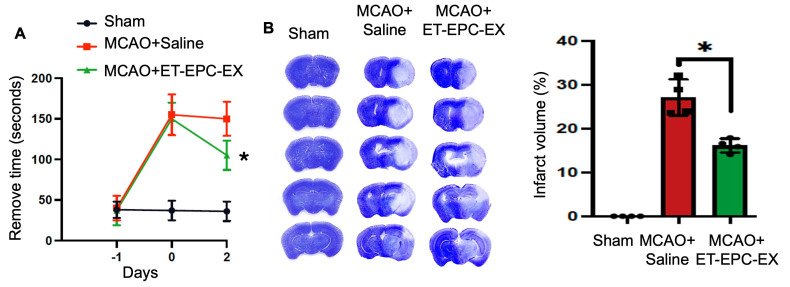
ET-EPC-EX improved the neuromotor function and reduced infarct size in hypertensive mice. (**A**) Sensorimotor deficits of ischemic mice were assessed by the adhesive removal test at different time points. *n* = 9 mice/group. * *p* = 0.04, vs. saline on day 2. Data was analyzed using two-way ANOVA followed by Tukey test. (**B**) Representative images and summarized data of Cresyl violet-stained coronal brain sections of sham, saline-treated, and ET-EPC-EX-treated mice 48 h after MCAO. *n* = 4 mice/group. * *p* = 0.0005, vs. saline. Data was analyzed using one-way ANOVA followed by Tukey test. Data was expressed as mean ± SD.

To further assess whether ET-EPC-EX can affect the motor function of the stroke mice, we then assessed the sensorimotor performance and infarct volume 48 h after MCAO. As shown in Figure 3A, in the adhesive removal test, all mice showed similar performance at baseline. After MCAO, the animals displayed marked deficits, with significantly prolonged removal time. Forty-eight hours later, the animals that received ET-EPC-EX treatment showed a clear trend towards faster removal times compared to saline controls (saline-treated mice: 150 ± 21 s; ET-EPC-EX-treated mice: 90 ± 18 s, *p* < 0.05, *n* = 8–9/group), suggesting partial functional rescue. Sham mice maintained relatively stable performance across all time points.

After the behavioral test, mice were transcardially perfused with ice-cold FBS and 4% PFA. The brains were then collected for histology analysis to determine the impact of ET-EPC-EXs at the tissue level. The infarct volume was assessed using CV staining. The data (Figure 3B) revealed large infarcts in saline-treated mice, whereas ET-EPC-EX treatment visibly reduced infarcted tissue across serial coronal sections. Quantification demonstrated a significant reduction in infarct volume in the ET-EPC-EX group compared with saline controls (*p* < 0.05, *n* = 4/group). Sham animals exhibited no infarction, as expected.

Together, these results indicate that early post-stroke administration of ET-EPC-EXs limits acute ischemic brain injury and supports improved early neurological recovery.

### 3.4. ET-EPC-EX Treatment Reduced Ischemia-Induced Cell Death in the Ipsilateral Hemisphere of Hypertensive Alb1 Mice

**Figure 4:**
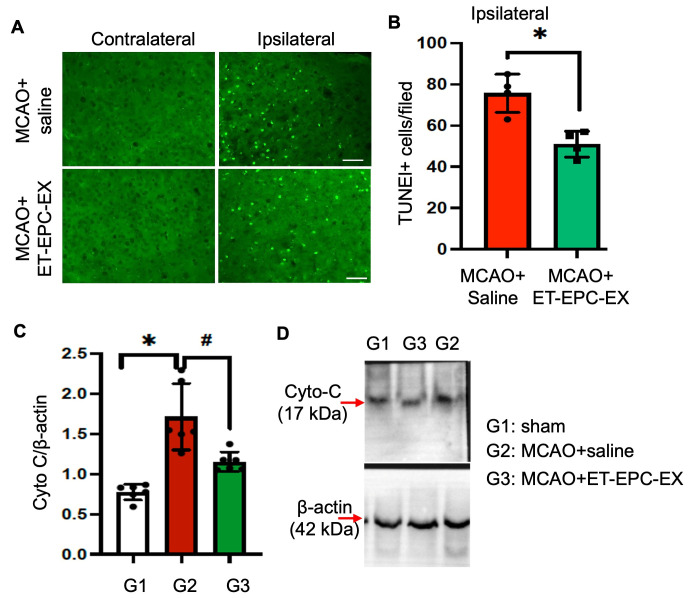
ET-EPC-EX reduced cell apoptosis in the ischemic brain. (**A**) Representative TUNEL staining in both contralateral and ipsilateral hemispheres at 48 h post-ischemia in both groups. (**B**) Summary data shows the quantification of TUNEL-positive cells per field. *n* = 4 mice/group. *p* = 0.006, vs. saline. t = 4.405, Cohen’s d is 5.29. Data was analyzed using an unpaired *t*-test. (**C**,**D**) Cyto-c protein level. *n* = 5 mice/group. * *p* < 0.0001; vs. sham; # *p* = 0.005; vs. saline. Data was analyzed using one-way ANOVA followed by the Tukey test. Data were expressed as mean ± SD.

To determine whether ET-EPC-EXs reduce ischemia-induced cell death, we quantified apoptotic burden using TUNEL staining forty-eight hours after MCAO. As shown in Figure 4A,B, saline-treated mice had more TUNEL-positive nuclei throughout the ipsilateral hemisphere, reflecting substantial cells undergoing apoptosis. In contrast, ET-EPC-EX-treated mice showed a marked reduction in TUNEL-positive cells. Quantification confirmed an approximately 32% decrease in apoptotic cell density relative to saline controls (*p* < 0.05; *n* = 4/group). Additionally, our data shows that the cytochrome c level was significantly reduced in the ipsilateral brain tissue of ET-EPC-EX-treated mice (Figure 4C,D). Together, these data demonstrate that ET-EPC-EXs mitigate cell death in the acute phase after stroke.

### 3.5. ET-EPC-EX Treatment Restored the Expression of Tight Junction Proteins After Ischemic Stroke

**Figure 5:**
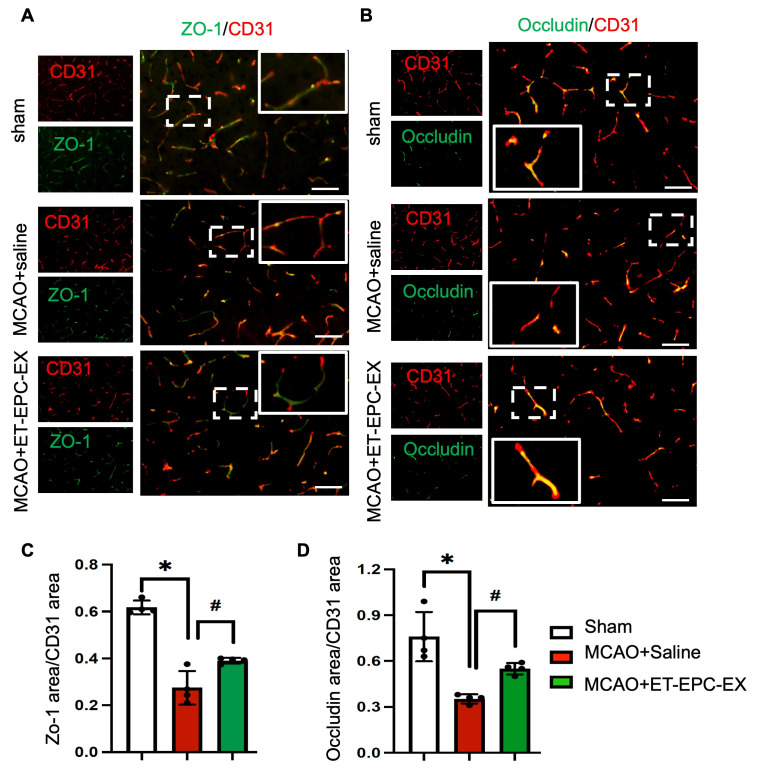
ET-EPC-EX treatment preserves endothelial tight-junction protein in the ischemic brain. (**A**) Representative immunofluorescence images of ZO-1 (green) colocalized with CD31^+^ endothelial cells (red) in the peri-infarct cortex. Scale bars: 20 μm. (**B**) Representative immunofluorescence images of occludin (green) colocalized with CD31^+^ endothelial cells (red) in the peri-infarct cortex. Scale bars: 20 μm. (**C**) Quantification of the percentage of ZO-1^+^ area normalized to CD31^+^ endothelial area. *n* = 4 mice/group; * *p* < 0.001; vs. sham; # *p* = 0.014, vs. saline; Data was analyzed using one-way ANOVA followed by Tukey test. (**D**) Quantification of the percentage of occludin^+^ area normalized to CD31^+^ endothelial area. *n* = 4 mice/group. * *p* = 0.033; vs. sham; # *p* = 0.043, vs. saline; Data was analyzed using one-way ANOVA followed by Tukey test. Data was expressed as mean ± SD.

The expression of tight junction proteins such as occludin and ZO-1 in the brain two days after ET-EPC-EX treatment was assessed. As shown in Figure 5A,B, in sham animals, ZO-1 (in green) and occludin (in green) showed strong and continuous co-localization (in orange) along CD31^+^ microvessels (in red; endothelial cell marker). In saline-treated mice, MCAO caused a marked loss of ZO-1 and occludin immunoreactivity, accompanied by fragmented or discontinuous staining along CD31^+^ microvessels. In contrast, ET-EPC-EX treatment substantially preserved both the intensity and continuity of tight junction labeling, with endothelial structures showing more intact morphology and co-localization with CD31. Quantitative image analysis demonstrated significant reductions in ZO-1^+^/CD31^+^ and occludin^+^/CD31^+^ co-localized areas in saline-treated mice, whereas ET-EPC-EX treatment restored these tight junction markers to levels towards those observed in sham animals (*p* < 0.05; Figure 5C,D).

Collectively, these results indicate that ET-EPC-EXs preserve BBB structural integrity after stroke by maintaining tight junction architecture and protecting endothelial cells from ischemic injury.

### 3.6. ET-EPC-EX Treatment Dampened Neuroinflammation in Hypertensive Alb1 Mice Following MCAO

**Figure 6:**
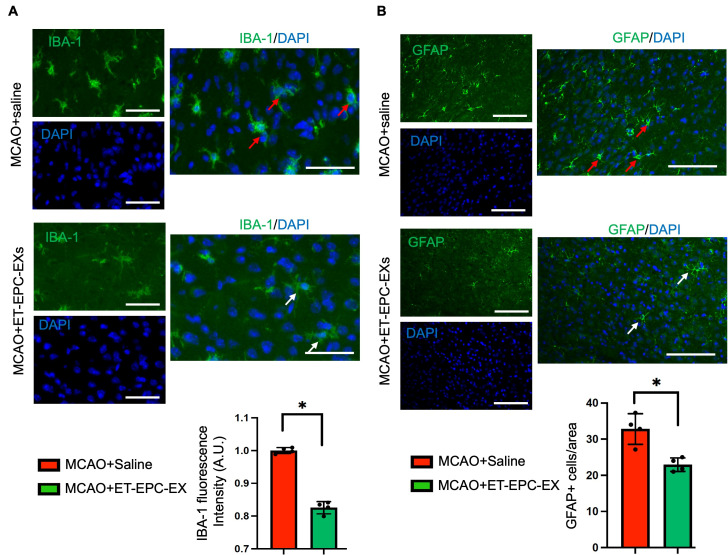
ET-EPC-EX treatment dampens neuroinflammation in the ischemic brain. (**A**) Representative immunofluorescence images and summary data of IBA-1 (green) in the ipsilateral hemisphere. Scale bars: 50 μm. Red arrow: reactive microglia with enlarged somas and thickened and retracted processes. White arrow: ramified microglia. *n* = 4 mice/group. * *p* < 0.001; vs. saline; t = 16.89, Cohen’s d is 16.2. Data was analyzed using unpaired *t*-test. (**B**) Representative immunofluorescence images and summary data of GFAP (green) in the ipsilateral hemisphere. Scale bars: 50 μm. Red arrow: reactive astrocytes with enlarged soma. White arrow: “resting” astrocyte. *n* = 4 mice/group. * *p* = 0.012; vs. saline; t = 4.27, Cohen’s d is 4.2. Data was analyzed using unpaired *t*-test. Scale bars: 50 μm. Data was expressed as mean ± SD.

Growing evidence suggests that the astrocyte- and microglia- mediatednflammatory response plays an important role in prognosis after acute ischemic stroke. According to the immunofluorescence data, we found that in saline-treated MCAO mice, microglia exhibited a pronounced reactive morphology characterized by enlarged somas and thickened and retracted processes (red arrow; Figure 6A), indicating robust neuroinflammatory activation. In contrast, in those received ET-EPC-EXs, the IBA-1 positive microglia exhibited a shift toward a more ramified morphology (white arrow; Figure 6A), resembling a “resting” and less inflammatory state. The IBA1 fluorescence intensity per area was significantly reduced in ET-EPC-EX-treated brains compared to saline-treated mice (*p* < 0.05; *n* = 4 mice/group; Figure 6A), showing a reduction in microglial activation.

In parallel, our data also showed that the number of GFAP^+^ cells per field in the ipsilateral hemisphere was lower in ET-EPC-EX-treated mice (22.64 ± 1.85 GFAP^+^ cells/field) than in saline-treated mice (32.82 ± 4.22, GFAP^+^ cells/field) (*p* < 0.05; *n* = 4 mice/group; Figure 6B), along with high expression of GFAP and enlarged soma size (red arrows; Figure 6B). This data suggests that ET-EPC-EX treatment alleviated the activation of astrocytes.

### 3.7. ET-EPC-EX Treatment Decreased MMP3 Level and Inactivated the P38 Signaling Pathways in the Ischemic Brain

**Figure 7:**
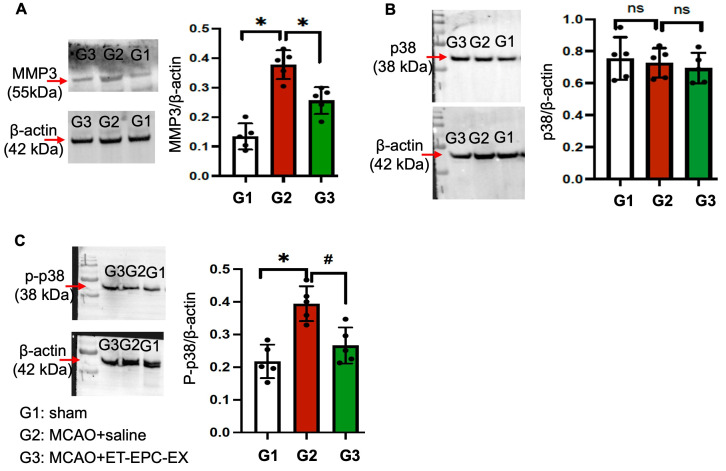
ET-EPC-EX treatment reduced MMP3 level and inactivated the p38 signaling in the ischemic brain. (**A**) MMP3 protein level in sham mice and mice treated with saline or ET-EPC-EXs. * *p* = 0.003; vs. sham or saline; *n* = 5 mice/group. (**B**) p38 level in sham mice and mice treated with saline or ET-EPC-EXs. *p* = 0.91, vs. sham; *p* = 0.88, vs. saline. *n* = 5 mice/group. ns: not significant. (**C**) p-p38 level in sham mice and mice treated with saline or ET-EPC-EXs. * *p* = 0.0006; vs. sham; # *p* = 0.006; vs. saline; *n* = 5 mice/group. Data was analyzed using one-way ANOVA followed by Tukey test. Data was expressed as mean ± SD.

The p38 MAPK signaling pathway is a major upstream regulator of post-ischemic cell loss. Whether p38 signaling can be altered across experimental groups is unknown; therefore, we evaluated the expression levels of total p38, phosphorylated p38 (p-p38), and MMP3 in the ischemic brain. Quantitative analysis revealed a significant increase in MMP3 expression in saline-treated mice compared with sham mice (*p* < 0.05; Figure 7A). This elevation was decreased in ET-EPC-EX-treated mice, which showed significantly lower MMP3 levels (*p* < 0.05; *n* = 5/group). The total p38 protein levels did not differ significantly among all experimental groups, indicating that p38 expression was not altered across conditions. However, phosphorylation of p38 was markedly increased in saline-treated mice (*p* < 0.05), which was significantly reduced by ET-EPC-EXs (*p* < 0.05; *n* = 5/group; Figure 7B,C). Together, these findings demonstrate that MMP3 expression was increased and the p38 signaling cascades were activated after MCAO. ET-EPC-EX treatment suppressed p38 phosphorylation and reversed MMP3 upregulation.

## 4. Discussion

In this study, we demonstrate that exercise-preconditioned EPC-EXs elicit robust benefits during the acute phase of ischemic stroke in hypertensive mice. Particularly, our findings show that ET-EPC-EXs counteract hypertension-driven vulnerabilities by preserving CBF, attenuating neuroinflammation and apoptosis, and stabilizing tight-junction proteins. Furthermore, we found that sensorimotor function was improved in ET-EPC-EX-treated mice compared to the saline-treated mice 2 days following MCAO. These results provide the first in vivo evidence that the exercise-conditioned EPC secretomes have therapeutic potential in hypertensive stroke.

Previous studies reported that hypertension impairs endothelial function and increases endothelial stiffness [28]. It downregulates tight-junction proteins (e.g., occludin, ZO-1) [29] and weakens BBB integrity [30], which exacerbates ischemic brain injury. Clinical and animal studies also revealed that hypertension compromises EPC function [31], impairing endogenous vascular repair capacity. Despite recent advances in thrombolysis and thrombectomy, hypertensive patients remain inadequately treated by current neuroprotective strategies. Exercise is one of the few interventions known to restore EPC activity by improving cell mobilization, survival, and paracrine signaling [32]. While many studies have explored exercise-induced neuroprotection, few have investigated the effects of specific circulating components such as EPC-EXs. This study provides the first direct evidence that ET-EPC-EXs can reproduce early protective effects under hypertensive conditions in vivo. Previous work has shown that EPC-EXs protect cardiomyocytes against hypertrophy and apoptosis [33] and possess enormous potential to treat the ischemic heart [34]. Our study extends these observations to hypertensive stroke, a condition in which cerebrovascular repair mechanisms are typically compromised. The current work fills a critical gap by demonstrating that ET-EPC-EXs retain therapeutic efficacy even in the context of chronic cerebrovascular dysfunction.

Many groups, including us, have demonstrated that EVs from EPCs have the potential to promote vascular repair by stimulating angiogenesis, supporting endothelial survival, and restoring microvascular integrity [26,35]. In models of ischemic stroke, EXs can efficiently cross the BBB and accumulate in injured brain tissues, enabling targeted delivery of reparative molecular cargo to reduce infarct size and improve functional recovery [26]. Consistent with our earlier findings that exercise conditioning enhances the reparative functions of EPC-EXs in C57 BL/6 mice [9], the present study demonstrates that ET-EPC-EXs confer robust brain protection in a hypertensive ischemic stroke mouse model. Specifically, ET-EPC-EX treatment significantly improved CBF recovery within 48 h after MCAO, indicating enhanced microvascular stability and early hemodynamic support. This beneficial effect aligns with previous reports showing that EPC-derived vesicles promote endothelial survival and support cerebrovascular repair following ischemic injury [13]. We also observed that the ET-EPC-EXs labeled with PKH-26 were accumulated in CD31^+^ microvessels within the ipsilateral hemisphere, supporting a direct vascular mechanism of action. Functionally, ET-EPC-EX administration led to significant improvements in sensorimotor behavior as revealed by a shorter removal time of the adhesive tape. Given that early neurological deficits in hypertensive MCAO are strongly influenced by neurovascular dysfunction and inflammatory injury, improved performance likely reflects preserved cerebrovascular integrity and reduced secondary neurodegeneration. These early benefits are noteworthy, as hypertension reduces the efficacy of many neuroprotective interventions and limits the brain’s capacity for repair.

Tight-junction loss is a hallmark of BBB disruption, particularly in the hypertensive brain, where chronic renin–angiotensin system activation and oxidative stress weaken endothelial integrity. In the present study, ET-EPC-EXs preserved the tight-junction proteins ZO-1 and occludin in the ipsilateral brain, indicating stabilization of the BBB. The internalization of ET-EPC-EXs by multiple neurovascular cell types, including neurons, astrocytes, and microglia, suggests broader effects on the neurovascular unit that may synergistically promote tissue stabilization. ET-EPC-EX treatment also reduced apoptotic cell death and alleviated microglial and astrocyte activation, demonstrating potent anti-apoptotic and anti-inflammatory effects. Cyto-c, an essential component of the electron transport system, resides normally in mitochondria, and its translocation to cytoplasm is pivotal in precipitating apoptosis via the mitochondrial pathway [36]. In the present study, our data show that ET-EPC-EXs robustly reduced Cyto-c levels, associated with reduced cell apoptosis as revealed by TUNEL staining. This is supported by other studies showing that inhibition of Cyto-c release can substantially reduce the infarct volume [37,38].

Previous studies have demonstrated that microglia are recruited to the site of infarction and undergo proliferation in response to ischemic injury, contributing to the expansion of the inflammatory response after stroke [39,40]. Microglia rapidly respond to ischemic injury by transitioning from a ramified, homeostatic morphology to an activated state characterized by enlarged somas and retracted, thickened processes. This morphological transformation is closely associated with exacerbated neuronal death and secondary injury. Indeed, saline-treated MCAO mice in our model exhibited pronounced microglial activation, as reflected by reactive morphology and increased IBA1 fluorescence intensity per field. In contrast, ET-EPC-EX treatment significantly promoted a shift toward a more ramified, less inflammatory microglial phenotype and reduced IBA1 immunoreactivity, indicating effective dampening of ischemia-induced microglial activation. Astrocytes play a complementary but equally critical role in shaping the post-ischemic inflammatory milieu. Reactive astrogliosis, marked by increased soma, GFAP expression, and astrocytic proliferation, can initially provide structural and metabolic support but often becomes maladaptive, contributing to glial scar formation, inflammatory amplification, and BBB dysfunction. Our observation shows that ET-EPC-EXs significantly reduced the percentage of GFAP^+^ cells in the ipsilateral hemisphere, suggesting that these vesicles can limit excessive astrocytic activation. The coordinated suppression of both microglial and astrocytic reactivity underscores the broad immunomodulatory capacity of ET-EPC-EXs in the ischemic brain.

Mechanistically, our data implicates the p38 MAPK-MMP-3 axis as a critical downstream pathway regulated by ET-EPC-EX treatment. The p38 signaling pathway is a mediator of ischemia-induced inflammation and cell death, particularly in hypertension-exacerbated stroke. Activation of p38 promotes transcription of inflammatory genes, mitochondrial dysfunction, and apoptosis, while also inducing expression of matrix metalloproteinases such as MMP-3 [16,17]. MMP-3 is predominantly expressed by reactive astrocytes and microglia after brain injury [41] and contributes to BBB disruption by degrading extracellular matrix components and endothelial tight junction proteins [42]. In line with these pathological roles, we observed marked upregulation of MMP-3 and increased p38 phosphorylation in saline-treated MCAO mice. Importantly, ET-EPC-EX treatment significantly suppressed p38 phosphorylation without altering total p38 protein levels, indicating inhibition of pathway activation rather than changes in protein expression. This was accompanied by a robust reduction in MMP-3 levels, suggesting that ET-EPC-EXs interfere with upstream inflammatory signaling that drives protease induction. Given the established role of MMP-3 in BBB breakdown and neurovascular injury, downregulation of the p38-MMP-3 cascade likely represents a key mechanism through which ET-EPC-EXs preserve neurovascular integrity and limit secondary damage after ischemic stroke. This is in line with the laser speckle imaging findings showing that ET-EPC-EXs significantly improved early CBF recovery after MCAO in live animals. These findings suggest that modulation of MAPK signaling may be a central mechanism through which ET-EPC-EXs exert cerebrovascular protection. While we identified p38 suppression as an associated pathway, specific p38 inhibitors will be applied in future studies to further determine whether the p38 pathway is a causative one.

Taken together, our findings support a model in which ET-EPC-EXs mitigate hypertensive ischemic brain injury by suppressing neuroinflammation and stabilizing BBB tight junction proteins, associated with the inhibition of the p38 pathway. These effects likely complement previously described actions of EPC-EXs on endothelial survival and vascular repair, highlighting their multifaceted therapeutic potential. By targeting both vascular and neuroinflammatory components of ischemic pathology, ET-EPC-EXs may be particularly well suited for stroke treatment in high-risk hypertensive populations.

There are some limitations in the present study. First, EXs were administered only at a single time point during the acute phase; dose–response or delayed-treatment windows have not been evaluated. Future studies will also include experiments to test the effects of ET-EPC-EXs in a wider therapeutic window, such as 12 h, to enhance translational application to treat hypertensive stroke patients in clinical settings. Second, long-term outcomes such as chronic inflammation and post-stroke cognitive impairment after 4 weeks or longer remain to be investigated. Third, future studies are needed to include a non-exercised exosome group to delineate the specific contribution of exercise conditioning and identify the specific exosomal cargos (such as miR-27a) mediating these observed effects.

## 5. Conclusions

In conclusion, our findings demonstrate that EPC-derived exosomes from exercised mice confer significant neurovascular protection in hypertensive ischemic stroke, as evidenced by improved CBF, stabilized BBB, reduced apoptosis, and attenuated neuroinflammation. These results support the therapeutic potential of EPC-derived exosomes as a cell-free strategy for acute hypertensive stroke.

## Data Availability

The raw data supporting the conclusions of this article will be made available by the authors on request.
